# The efficacy and safety of cilostazol for the secondary prevention of ischemic stroke in acute and chronic phases in Asian population- an updated meta-analysis

**DOI:** 10.1186/s12883-014-0251-7

**Published:** 2014-12-20

**Authors:** LiGen Shi, JiaLi Pu, Liang Xu, Jay Malaguit, Jianmin Zhang, Sheng Chen

**Affiliations:** Department of Neurosurgery, Second Affiliated Hospital, School of Medicine, Zhejiang University, 88 Jiefang Road, Hangzhou, 310009 Zhejiang China; Department of Neurology, Second Affiliated Hospital, School of Medicine, Zhejiang University, Hangzhou, Zhejiang China; Department of Physiology and Pharmacology, Loma Linda University, Loma Linda, CA USA

**Keywords:** Acute Phase, Chronic phase, Cilostazol, Meta-analysis, Stroke

## Abstract

**Backgrounds:**

While previous meta-analysis have investigated the efficacy of cilostazol in the secondary prevention of ischemic stroke, they were criticized for their methodology, which confused the acute and chronic phases of stroke. We present a new systematic review, which differs from previous meta-analysis by distinguishing between the different phases of stroke, and includes two new randomized, controlled trials (RCTs).

**Methods:**

All RCTs investigating the effect of cilostazol on secondary prevention of ischemic stroke were obtained. Outcomes were analyzed by Review Manager, including recurrence of cerebral infarction (ROCI), hemorrhage stroke or subarachnoid hemorrhage (HSSH), all-cause death (ACD), and modified Rankin Scale score (mRS). The Grading of Recommendations Assessment, Development and Evaluation (GRADE) assessed the quality of the evidence.

**Results:**

5491 patients from six studies were included in the current study. In secondary prevention of ischemic stroke in chronic phase, cilostazol was associated with a 47% reduction in ROCI (relative risk [RR] 0.53, 95% confidence interval [CI] 0.34 to 0.81, *p* = 0.003), while no significant difference in HSSH and ACD compared with placebo; and 71% reduction in HSSH (RR 0.29, 95% CI 0.15 to 0.56, *p* = 0.0002) compared with aspirin, but not in ROCI and ACD. In the secondary prevention of ischemic stroke in acute phase, cilostazol did not show any effect in the ROCI, HSSH, ACD and mRS compared to placebo or aspirin. The quality of the evidence from chronic phase was high or moderate, and those from acute phase were moderate or low when analyzed by GRADE approach.

**Conclusion:**

Cilostazol provided a protective effect in the secondary prevention of the chronic phase of ischemic stroke.

## Background

Stroke accounts for 10% of all deaths worldwide [1], and is the second leading cause of mortality in China [2]. Recurring strokes in approximately 30% of patients showed more severe consequences than primary stroke, usually leading to dementia and death [3]. Thus, secondary prevention in high-risk patients with a previous stroke becomes extremely important. Among the various medical managements, aspirin plays a pivotal role in the secondary prevention of stroke because of its antiplatelet efficacy [4]. However, this efficacy has prominent race-ethnic differences, a recent study reported that Asians have a higher risk of recurrent ischemic and hemorrhagic stroke in the secondary stroke prevention phase [5]. Besides systemic hemorrhagic events, common side effects including dyspepsia, abdominal pain, and gastric ulcers have also limited its clinical application [6,7]. It is therefore necessary to develop an effective new drug with a lower incidence of side effects for aspirin intolerant populations. Cilostazol, an antiplatelet agent with selectively inhibiting phosphodiesterase III, is such an agent [8]. It not only prevents the inactivation of intracellular cyclic adenosine monophosphate (cAMP) and inhibits platelet aggregation, but also improves endothelial function and inhibits the proliferation of arterial smooth muscle cells [9]. Furthermore, several randomized controlled trials (RCTs) indicate that cilostazol had lessintracranial hemorrhage risks, compared with aspirin in the secondary prevention of the stroke [2,4,9,10].

Previous systematic reviews and meta-analysis of RCTs determined whether cilostazol reduces morbidity and mortality compared with aspirin for the secondary prevention of stroke [11,12]. However, these meta-analysis were incomplete in several respects. The major criticism was its methodology that confused the acute and chronic phases of stroke. It has been confirmed that the acute phase of reperfusion injury exists in animal models of ischemic stroke, which plays an important role in the microcirculation levels [13]. It has also been confirmed that 20% to 40% of all patients in acute phase have aprogressive worsening of clinical and/or radiologic features even with currently available treatments [14]. Furthermore, recent studies have demonstrated that cilostazol might be a protective agent in the secondary prevention of the chronic phases of stroke [2,4,9,15]. But the benefit of cilostazol in the prevention of stroke was controversial [10,16]. The minor criticisms contributing to the unsoundness of previous meta-analysis included the lack of data pertaining to the efficacy of cilostazol compared with a placebo. A recent RCT reported negative results of cilostazol against acute progressing stroke [16], which differed from a previous RCT [10] and might reverse the conclusion of the previous meta-analysis. Hence, we present a new systematic review, which differs from the previous systematic reviews in their methodology and inclusion of another two new RCTs. Moreover, the Grading of Recommendations Assessment, Development and Evaluation (GRADE) approach was applied to assess the quality of the evidence [17-19].

## Methods

### Study protocol

At the beginning of this project, a study protocol was drafted following the Cochrane Collaboration format [20].

### Eligibility criteria

The present systematic review only included studies which met the following criteria: 1) study type: RCTs; 2) language restriction: only English studies were reviewed; 3) participants: adult patients suffered ischemic stroke; 3) intervention: cilostazol; 4) comparator: aspirin or placebo; 5) outcomes: recurrence of cerebral infarction (ROCI), hemorrhage stroke or subarachnoid hemorrhage (HSSH), and all-cause death (ACD). Exclusion criteria: 1) study types: case control study, cohort study, and retrospective study; 2) withdraw rate: > 20%; 3) participants: < 18 years.

### Search strategy and information sources

Two of the authors (LGS and JLP) independently searched the Medline database up to March 2014 for the combination of the variables “cilostazol” AND “stroke”. The search was limited to clinical studies and matched the titles and abstracts of studies. Moreover, we searched for all relevant RCT or meta-analysis studies in the Embase, Cochrane Library and the Cochrane Central Register of Controlled Trials published between Jan 1980 and March 2014. To insure all relevant studies had been included in this systematic review, besides the electronic database search, reference lists from RCTs and systematic reviews were manually screened. The appendices include details of the search strategies (Appendix).

### Study selection and data collection

We included RCTs that assessed the efficacy and acceptability of cilostazol compared with placebo or aspirin treatment in patients with the history of stroke, as diagnosed by computed tomography (CT) or magnetic resonance imaging (MRI). All patients were recruited with the results for the secondary prevention of ischemic stroke in acute and chronic phases as separate subgroups. The acute phase of ischemic stroke was diagnosed when the patient suffered a cerebral infarction within 48 hours prior to participation in the trial without serious complications, and the chronic phase was defined from 1 to 6 months. The outcomes included as the following: ROCI, HSSH, ACD, and modified Rankin Scale score (mRS), all based on intention-to-treat datasets.

After reading all included RCT articles, we extracted the following data, which were described in all studies: country, single or multiple therapeutic centers, inclusion criteria for the participants, general information of the patients (age and gender), and therapeutic schedule (usage of the drugs, doses, and duration). The four outcomes were also selected from each trial.

### Risk of bias and quality assessment

The Cochrane Collaboration tool was used in this systematic review to assess the risk of bias in each included RCT study. Two review authors (LGS and JLP) were independently assessed for methodological quality by the following six items: random sequence generation and allocation concealment (selection bias), blinding of participants and personnel (performance bias), blinding of outcome assessment (detection bias), incomplete outcome data (attrition bias), selective reporting (reporting bias) and other potential biases. For each item, the table provides a description and judgment rated as “low”, “unclear” or “high” risk of bias. The risk of bias plot was created using the Review Manager 5.2 software. GRADE approach was used to assess the quality of the evidence. In this approach, we mainly assessed five items, including risk of bias, inconsistency, indirectness, imprecision and publication bias, which can affect the quality of evidence. For each item, the table provides a judgment criteria rated as “high, moderate, low or very low”. After assessment of the evidence, GRADE pro 3.6 software created an evidence profiletable.

### Summary measures and synthesis of results

Data was processed in Review Manager 5.2 from the Cochrane Collaboration. Dichotomous outcomes were analyzed as the risk ratio (RR; 95% confidence interval [CI]) using the Mantel–Haenszel technique and a fixed effect model. Statistical heterogeneity was estimated by the *I*^*2*^statistic as follows: *I*^*2*^ < 30% means “low heterogeneity”, *I*^*2*^ = 30% to 50% denotes “moderate heterogeneity”, and *I*^*2*^ > 50% represents “substantial heterogeneity”. Tests were two-tailed and a *p* value less than 0.05 was considered to be significant for all analysis.

## Results

**Figure 1:**
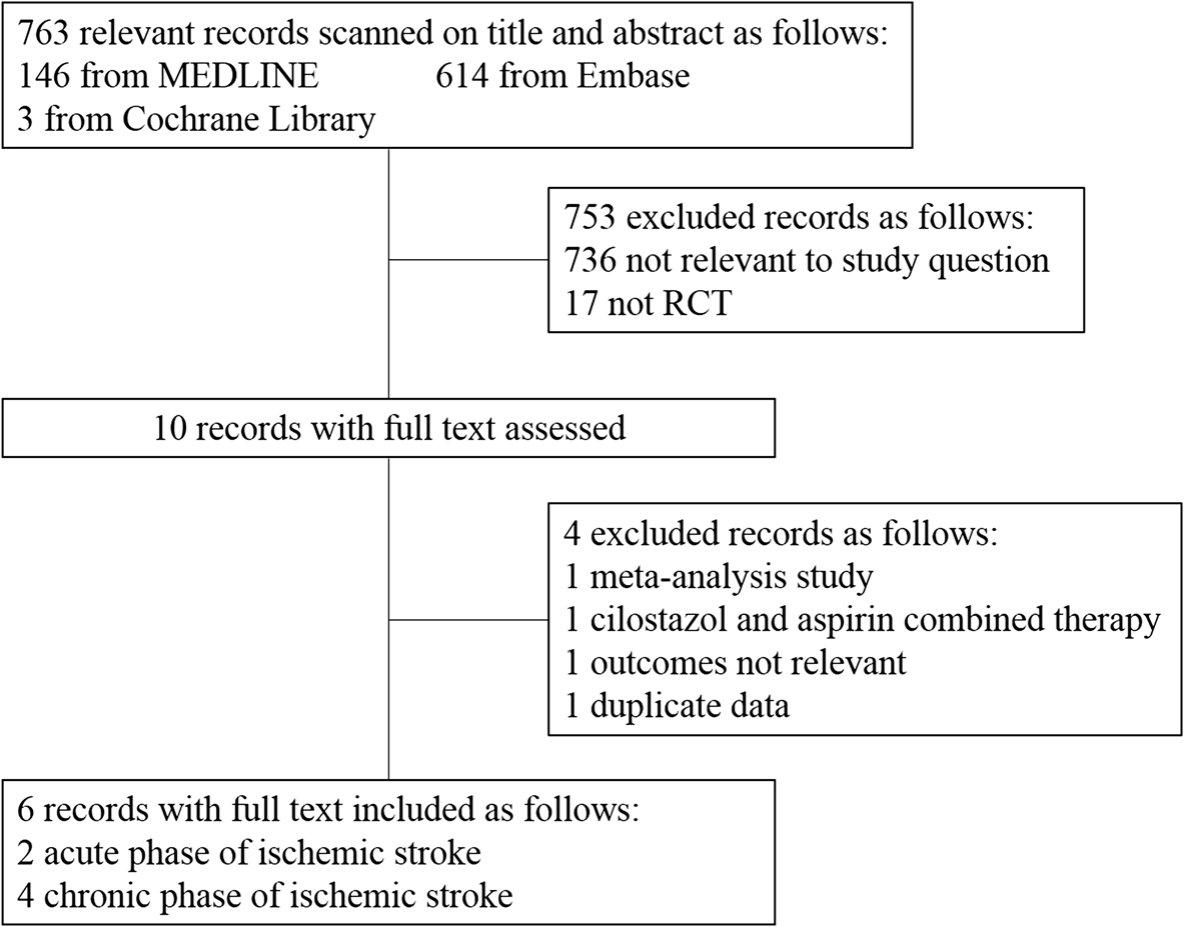
**Study search, selection and inclusion process.**

We retrieved 763 records after the initial search strategy that scanned for title and abstract. A further 753 records were excluded either for unrelated to the study question or not a RCT, resulting in 10 papers for further assessment. Another 4 records have been excluded from this analysis for the following reasons: meta-analysis record, cilostazol and aspirin combined therapy, irrelevant outcomes, and duplicate data. Finally, six RCTs on the basis of the inclusion criteria (Figure 1) were included with a total of 5491 patients.

### Study characteristics

The main characteristics and outcome events of the included RCT studies are listed in Table 1. The six RCTs, combined, enrolled 5491 patients including 965 patients in acute phase and 4526 patients in chronic phase of ischemic stroke. All the patients come from an Asian background, such as Japan, China, and Korea. The age of patients ranged from 59.4 to 66.6 years old. The follow-up time for the acute phase of ischemic stroke was 3 months, with the follow-up time for the chronic phase ranging from 1 to 5 years.

**Table 1:** **Characteristics of the included studies and outcome events**

**Articles**	**Country**	**Therapeutic centre**	**Inclusion criteria**	**Drugs**	**Age**	**Male percentage (%)**	**Dose**	**ITT population**	**Duration**	**ROCI %**	**HSSH %**	**mRS 0–1%**	**ACD %**
**Gotoh 2000**	Japan	183 clinical institutes	Cerebral infarction at 1 to 6 months	Cilostazol	65.2 (NC)	64.6	100 mg twice daily	533/1067	1-5 years	5.7	0.8	/	1.7
				Placebo	65.1 (NC)	60.8	NC	534/1067		10.8	1.3	/	1.9
**Huang 2008**	China	Multiple center trial	Cerebral infarction at 1 to 6 months	Cilostazol	60.14 (10.05)	66.9	NC	360/719	1-1.5 years	3.1	0.3	/	0.8
				Aspirin	60.31 (9.71)	70.5	NC	359/719		4.2	1.9	/	1.4
**Guo 2009**	China	Single center trail	Cerebral infarction at 1 to 6 months	Cilostazol	59.44 (10.63)	35.3	100 mg twice daily	34/68	1 year	5.9	0	/	0
				Aspirin	62.06 (11.12)	35.3	100 mgonce daily	34/68		2.9	2.9	/	5.9
**Shinohara 2010**	Japan	278 clinical institutes	Cerebral infarction in the previous 26 weeks	Cilostazol	63.5 (9.2)	71.7	100 mg twice daily	1337/2672	1–5 years	5.4	0.7	/	1.0
				Aspirin	63.4 (9.0)	71.7	81 mg once daily	1335/2672		6.6	2.3	/	1.0
**Lee 2011**	Korea	12 clinical institutes	Cerebral infarction within 48 h of onset	Cilostazol	63 (12)	64.1	100 mg twice daily	231/458	90 days	2.2	0	56.3	0.4
				Aspirin	63 (12)	58.6	300 mg/day	227/458		4.0	0.9	56.8	0
**Shimizu 2013**	Japan	55 clinical institutes	Cerebral infarction within 48 h of onset	Cilostazol	66.2 (9.4)	65.7	100 mg twice daily	251/507	90 days	1.2	0.8	74.5	0
				Placebo	66.6 (8.9)	68.4	NC	256/507		1.6	0.8	72.7	0

**ITT:** intention to treat; **ROCI:** Recurrence of Cerebral Infarction; **HSSH:** Hemorrhage Stroke or Subarachnoid Hemorrhage; **mRS:** modifiedRankin Scale; **ACD:** All Case Death; **NC:** Not Clear.

### Outcomes analysis

For all analysis pertaining to efficacy and acceptability, no evidence exists for the between-study of heterogeneities assessed by Cochrane *I*^*2*^ statistic (data not shown). No significant publication bias was shown in the funnel plots (data not shown).

#### The efficacy and safety of cilostazol in chronic phase

**Figure 2:**
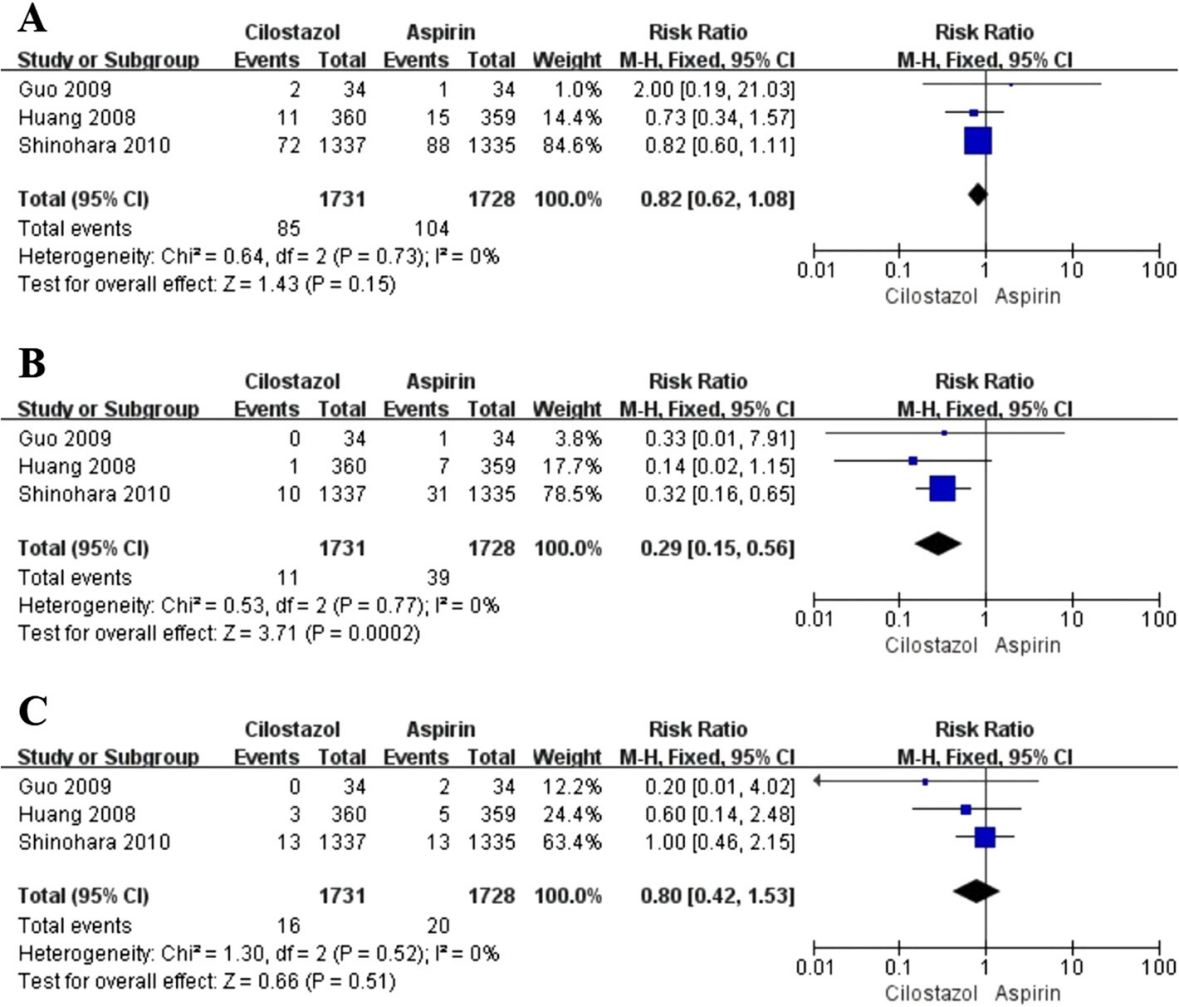
**Pooled relative risk of stroke recurrence,hemorrhagic stroke and all cause death between cilosatzol and aspirin groups insecondary prevention of stroke.** **(A)** Pooled relative risk estimates on recurrence of cerebral infarction. **(B)** Pooled relative risk estimates on hemorrhage stroke or subarachnoid hemorrhage. **(C)** Pooled relative risk estimates on all cause death. The diamond indicates the estimated relative risk (95% confidence interval) for all patients together.

Results for this analysis and the quality of this evidence were presented in Table 2. For the analysis of the efficacy and safety of cilostazol in chronic phase, all 4526 patients from all 4 studies were available (1067 patients from 1 study randomized to cilostazol or placebo, and 3459 patients from 3 studies to cilostazol or aspirin). In the placebo-controlled study, cilostazol therapy reduced the ROCI by 47% (RR 0.53, 95% CI 0.34 to 0.81, *p* = 0.003), and showed similar incidence in the HSSH (RR 0.57, 95% CI 0.17 to 1.94, *p* = 0.37) and ACD (RR 0.90, 95% CI 0.37 to 2.20, *p* = 0.82). These data were not shown in the tables or figures. In the aspirin-controlled studies, cilostazol therapy was associated with an insignificant 18% reduction in the ROCI (RR 0.82, 95% CI 0.62 to 1.08, *p* = 0.15; Figure 2A), and a 71% reduction in the HSSH (RR 0.29, 95% CI 0.15 to 0.56, *p* = 0.0002; Figure 2B) with no significant difference in the ACD (RR 0.80, 95% CI 0.42 to 1.53, *p* = 0.51; Figure 2C). In order to detect whether the consolidated results were influenced by one study with a large population [9], we performed the sensitivity analysis to confirm that the results were stable (Table 2).

**Table 2:** **Analysis and quality of the evidence using GRADE for efficacy and safety outcomes**

**Outcomes**	**No of participants (studies)**	**Relative effect (95% CI)**	**Risk of bias**	**Inconsistency**	**Indirectness**	**Imprecision**	**Publication bias**	**Quality of the evidence (GRADE)**
**1. Subgroup Analysis - Cilostazol compared to Aspirin for the Secondary Prevention of Stroke in the Chronic phase**								
**ROCI**	3459 (3 studies)	RR 0.82 (0.62 to 1.08)	No serious	No serious	No serious	No serious	Undetected	⊕ ⊕ ⊕ ⊕ **high**
**HSSH**	3459 (3 studies)	RR 0.29 (0.15 to 0.56)***	No serious	No serious	No serious	No serious	Undetected	⊕ ⊕ ⊕ ⊕ **high**
**ACD**	3459 (3 studies)	RR 0.80 (0.42 to 1.53)	No serious	No serious	No serious	No serious	Undetected	⊕ ⊕ ⊕ ⊕ **high**
**2. Sensitivity Analysis - Cilostazol compared to Aspirin for the Secondary Prevention of Stroke in the Chronic phase without CSPS 2 trial**								
**ROCI**	787 (2 studies)	RR 0.81 (0.40 to 1.66)	Serious^1^	No serious	No serious	No serious	Undetected	⊕ ⊕ ⊕⊝ **moderate**
**HSSH**	787 (2 studies)	RR 0.18 (0.03 to 0.99)*	Serious^1^	No serious	No serious	No serious	Undetected	⊕ ⊕ ⊕⊝ **moderate**
**ACD**	787 (2 studies)	RR 0.47 (0.13 to 1.64)	Serious^1^	No serious	No serious	No serious	Undetected	⊕ ⊕ ⊕⊝ **moderate**
**3. Sensitivity Analysis - Cilostazol compared to Aspirin for the Secondary Prevention of Stroke in the Chronic phase without Guo-2009 trail**								
**ROCI**	3391 (2 studies)	RR 0.80 (0.61 to 1.07)	No serious	No serious	No serious	No serious	Undetected	⊕ ⊕ ⊕ ⊕ **high**
**HSSH**	3391 (2 studies)	RR 0.29 (0.15 to 0.56)***	No serious	No serious	No serious	No serious	Undetected	⊕ ⊕ ⊕ ⊕ **high**
**ACD**	3391 (2 studies)	RR 0.89 (0.45 to 1.73)	No serious	No serious	No serious	No serious	Undetected	⊕ ⊕ ⊕ ⊕ **high**

ROCI: Recurrence of Cerebral Infarction; HSSH: Hemorrhage Stroke or Subarachnoid Hemorrhage; ACD: All Case Death; CI: Confidence Interval; RR: Risk Ratio; *P < 0.05; ***P < 0.001.

^1^Potential bias because of unclear of blinding.

GRADE Working Group grades of evidence:

High quality: Further research is very unlikely to change our confidence in the estimate of effect.

Moderate quality: Further research is likely to have an important impact on our confidence in the estimate of effect and may change the estimate.

Low quality: Further research is very likely to have an important impact on our confidence in the estimate of effect and is likely to change the estimate.

Very low quality: We are very uncertain about the estimate.

#### The efficacy and safety of cilostazol in acute phase

For the analysis of the efficacy and safety of cilostazol in acute phase, 965 patients from 2 studies were included (507 patients from 1 study randomized to cilostazol or placebo, and 458 patients from 1 study to cilostazol or aspirin). In the placebo-controlled study, cilostazol therapy showed no significant efficacy in the ROCI (RR 0.76, 95% CI 0.17 to 3.38, *p* = 0.72), and a similar result in the HSSH (RR 1.02, 95% CI 0.14 to 7.18, *p* = 0.98). In the aspirin-controlled study, cilostazol therapy was associated with no significant efficacy in the ROCI (RR 0.55, 95% CI 0.19 to 1.60, *p* = 0.27), and showed the similar incidence in the HSSH (RR 0.20, 95% CI 0.01 to 4.12, *p* = 0.30) and ACD (RR 2.95, 95% CI 0.12 to 72, *p* = 0.51). No significant difference existed between cilostazol therapy and placebo or aspirin groups in the mRS (data not shown).

### Risk of the bias

**Figure 3:**
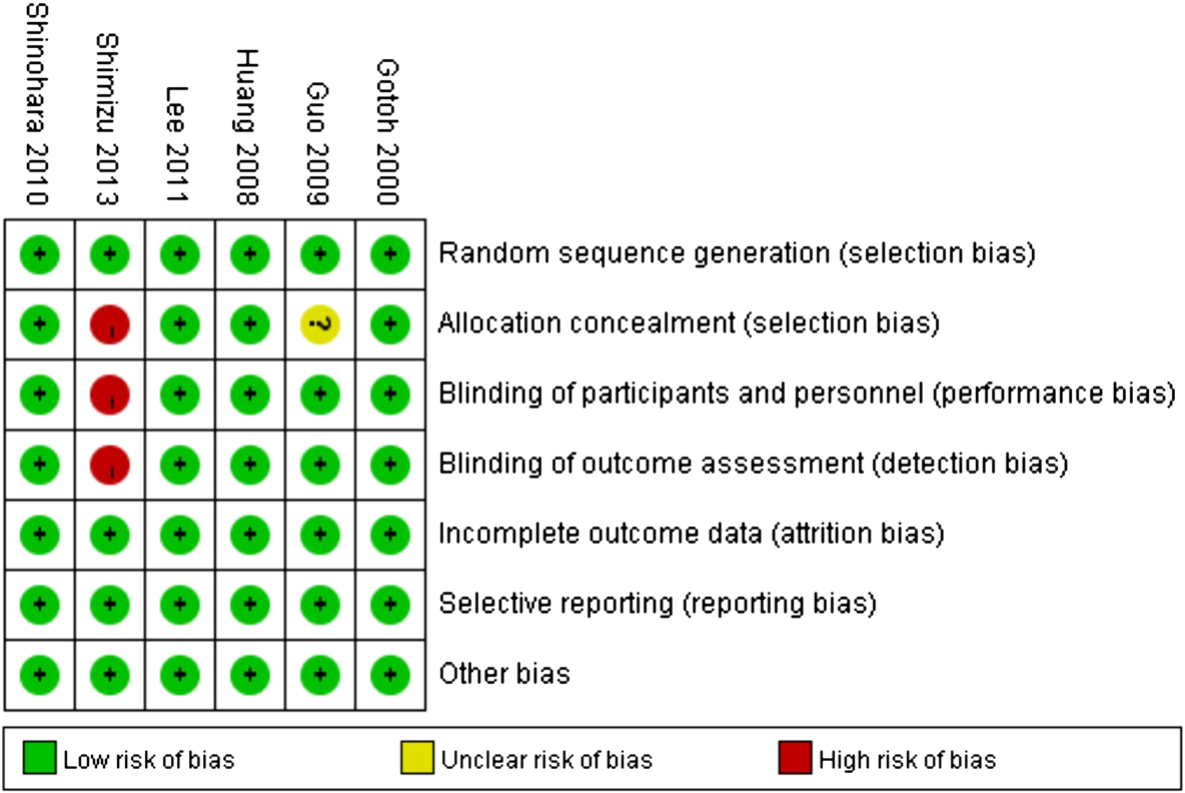
**Risk of bias: a summary table for each risk of bias items for each study.**

For allocation concealment, risk of bias was high in one study [16] and unclear in another one [4]. For blinding of participants and personnel, risk of bias was high in one study [16]. For blinding of outcomes assessment, risk of bias was high in one study [16]. Except these four items, no high risk of bias was observed in any of the other items (Figure 3).

## Discussion

The present systematic review and meta-analysis, including 6 RCTs in 5491 patients, evaluated the efficacy and acceptability of cilostazol for the secondary prevention of ischemic stroke. Our results suggest that cilostazol therapy leads to a significant reduction in ROCI compared to placebo, and a lower incidence of the HSSH compared with aspirin in the chronic phase. While in acute phase, cilostazol showed no better efficacy in the ROCI than placebo, and had a similar incidence of the HSSH to aspirin therapy. These findings support that cilostazol may be an advisable therapeutic alternative for aspirin in the secondary prevention of the chronic phase of ischemic stroke. Quality of the evidence for the outcomes from chronic phase studies assessed by GRADE was high or moderate, which provides a sufficient confidence at the application of cilostazol in clinical practice.

Compared with the methodology of the previous meta-analysis [11,12], the present systematic review explored the scope of cilostazol application for the secondary prevention of ischemic stroke. First, we defined the acute and chronic phases of ischemic stroke according to the onset-to-treatment time as follows: i) acute phase refers to patients having had an ischemic stroke within the preceding 48 hours; ii) the definition of chronic phase refers that patients were enrolled 1 to 6 months after ischemic stroke. The previous meta-analysis [11,12], did not distinguish between the acute and chronic phases. The present systematic review showed that cilostazol had controversial efficacy in the prevention of acute ischemic stroke. It is mainly due to the starting treatment time after onset. Many of the previous studies have reported that approximately 20%-37% of patients with acute ischemic stroke worsened gradually or stepwise after onset [21,22]. Among these patients, 58%-82% deteriorated progressively during the first 24 hours [23-25]. Hence, patients included within the first 24 hours of the Shimizu et al. [10] study may have progressed to a worse condition, which influenced the efficacy of cilostazol. In cases relating to thechronic phase of ischemic stroke, cilostazol showed a significant beneficial effect, which was consistent with the previous studies [2,4,9,11,12]. Second, in the previous meta-analysis [11,12], only aspirin-controlled RCTs were included to assess the efficacy of the cilostazol, which concluded that no significant difference existed in ROCI between cilostazol and aspirin. In the present systematic review, we have included both aspirin and placebo-controlled RCTs, which resulted in a more comprehensive conclusion that cilostazol has a definite effect in the ROCI, but not better than aspirin. Furthermore, we concluded that cilostazol is safer than aspirin in the HSSH, which was similar to the placebo. Third, we used a fixed-effects approach in meta-analysis where data did not indicate heterogeneity.

Current stroke guidelines from the American Stroke Association (ASA) and American Heart Association (AHA), recommend aspirin, clopidogrel, or aspirin plus extended-release dipyridamole as first-line options for secondary prevention of ischemic events (Class IIa, Level of Evidence A) [26]. Aspirin has a wide dose range from 50 to 1300 mg/d to prevent the reoccurrence of stroke [27]. However, both high- or low-dose aspirin may cause intracranial hemorrhagic events, which could limit its clinical application [28]. Cilostazol, a novel antiplatelet drug, prevents the recurrence of ischemic stroke through its antiplatelet effects [8], vasodilation, inhibition of vascular smooth muscle cell growth, and neuroprotection. Several randomized, multicentered trials demonstrated the preventable effect of cilostazol in patients with a previous stroke. According to the Cilostazol Stroke Prevention Study, patients with a prior stroke, who were allocated to cilostazol 100 mg twice daily or placebo, showed that cilostazol therapy reached a significant 58.3% reduction in ROCI, with no clinically significant adverse reactions [15]. Three other RCTs compared with aspirin show that cilostazol not only had similar therapeutic effects with aspirin in the ROCI, but also had a significant reduction in the HSSH [2,4,9]. In the animal studies, cilostazol showed a better effect than aspirin in the reduction of brain damage after ischemic stroke though suppressing disruption of the microvasculature and increasing the residual perfusion of microcirculation [29,30]. A pilot study, reported that patients treated with combined therapy had less neurological deterioration and a more favorable functional status than those treated with aspirin alone in secondary prevention of acute ischemic stroke [31]. Another double-blind multicenter trial containing 244 aspirin subjects with ischemic stroke who were randomly assigned to receive cilostazol 100 mg twice daily or placebo, observed a trend toward enhanced antiplatelet effects when cilostazol was added to aspirin in ischemic stroke patients [32]. It should be noted however, that cilostazol was more likely to cause several adverse events other than intracranial hematoma in comparison with aspirin [9]. The most common adverse events were put in descending order of occurrence as follows: headache, diarrhea, palpitations, dizziness, and tachycardia [9]. These findings supported that cilostazol is an alternative drug of aspirin but still needs large, randomized, multicentered trials to confirm the efficacy and safety of cilostazol.

In the present review, several factors may affect the combining of data, despite the statistics showing a low heterogeneity. For the treatment duration, 2 studies [9,15] followed up for 1–5 years, while another two studies [2,4] were 1–1.5 years. However, this difference in the date of treatment may only play a minor role because the Kaplan-Meier curves for the accumulation of primary endpoints showed a steady trend after 400 days [2]. For the stroke etiologies, the most common type was lacunar infarction followed by atherothrombotic infarction in the included studies. Three studies provided similar findings in about 65% -75% proportion of lacunar infarction, and no significant difference existed between cilostazol and controlled groups [9,15,16]. Another three studies lacked data regarding stroke etiologies [2,4,10], which might affect the combining of data to some degree. For the different vascular risk factors, a previous review has indicated that hypertension, diebetes, and hyperlipidemia maybe the main causes to influence the efficacy of secondary prevention [33]. In the present review, all six included studies recruited approximately 70% hypertension, 30% diebetes, and 30% hyperlipidemia. Only one study showed significant differences in systolic blood pressure between cliostazol and controlled groups [9]. Although the authors clarified that no interaction existed between treatment group and measurement time-points for systolic or diastolic blood pressure, the results of sensitivity analysis, without this study, showed that the difference became smaller but did not reverse the results (Table 2).

Several limitations of the present study should be considered. First, the present meta-analysis only included 4 studies for the chronic phase of ischemic stroke, and 2 studies for acute stroke. The number of studies is small, which may cause reporting bias. The result of secondary prevention for chronic phase of ischemic stroke was based on one placebo-controlled RCT, which was not an effect size. This same limitation also existed in the results of acute phase. Caution should be used when applying these results in the clinical setting. Secondly, not all of the included studies were double-blind, randomized, controlled trails. The Cilostazol for the Prevention of Acute Progressing Stroke was an open, multicenter, randomized controlled trial [16]. The information of blinding was not available from the study of Guo et al. [4], which made it difficult to determine if this trial was double-blinded. Finally, all of the patients from the included studies were of Asian descent, suggesting a limited confidence when applying this data to other populations. While aspirin was an ideal option for the secondary prevention of stroke in western countries, it did not seem suitable for those of Asian descent [5]. Previous studies have reported that Asians are at a higher risk forside effects including recurrent ischemic and hemorrhagic strokes in the secondary stroke prevention phase [5]. Genetic factors in different race-ethnicities, as independent predictors of cerebrovascular disease, maybe the main reason for high risk of side effects in Asian population [34-36]. In addition, higher and poorer control of blood pressure in Asians may be another factor contributing to the poor efficacy of aspirin in secondary stroke prevention [5]. Hence, cilostazol might be a safer option for Asians because of its reduced risk of intracerebral hemorrhage when compared to aspirin. All of these limitations were considered in the evaluating the quality of evidence.

## Conclusion

Cilostazol therapy played a crucial role in the secondary prevention of ischemic stroke in chronic phase. No significant difference was presented in ROCI between cilostazol and aspirin, but cilostazol was deemed to be safer. Prospective large RCTs will provide more evidence for cilostazol as an alternative drug for aspirin in secondary prevention of stroke.

## Appendix

1. (cilostazol [Title/Abstract] OR pletal [Title/Abstract] OR pletaal[Title/Abstract] OR OPC 13013 [Title/Abstract] OR OPC 21 [Title/Abstract])
2. (aspirin [Title/Abstract] OR acetylsalicylic acid [Title/Abstract] OR acetyl salicylic acid[Title/Abstract] OR acetosalicylic acid [Title/Abstract] OR placebo [Title/Abstract])
3. (ischemi* [Title/Abstract] OR stroke [Title/Abstract] OR cerebrovascular [Title/Abstract] OR intracerebral [Title/Abstract] OR embolism [Title/Abstract] OR thrombosis [Title/Abstract])
4. 1 AND 2 AND 3
5. Limit 4 to humans
